# Effect of the Rare Earth Element Lanthanum (La) on the Growth and Development of Citrus Rootstock Seedlings

**DOI:** 10.3390/plants10071388

**Published:** 2021-07-06

**Authors:** Hang Yin, Junxiu Wang, Yao Zeng, Xinjian Shen, Yizhong He, Lili Ling, Li Cao, Xingzheng Fu, Liangzhi Peng, Changpin Chun

**Affiliations:** Citrus Research Institute, Southwest University, Chongqing 400700, China; 13110283789@163.com (H.Y.); 15730032182@163.com (J.W.); 18789027757@163.com (Y.Z.); sxj123@163.com (X.S.); heyizhong@cric.cn (Y.H.); linglili@cric.cn (L.L.); caoli@cric.cn (L.C.); fuxingzheng@cric.cn (X.F.); pengliangzhi@cric.cn (L.P.)

**Keywords:** citrus rootstock, REEs, La, growth and development

## Abstract

Rare earth elements (REEs) can affect the growth and development of plants. However, few studies have been carried out on the effects of REEs on citrus seedlings. In this study, the growth parameters, toxicity symptoms, chlorophyll content, and La content of three citrus rootstocks are analyzed under different concentrations of La, a representative REE. The results show that the growth of citrus rootstock seedlings was stimulated at La ≤ 0.5 mmol·L^−1^ and inhibited at concentrations above 1 mmol·L^−1^. The chlorophyll and carotenoid contents of trifoliate orange (*Poncirus trifoliata* L. Raf.) and Ziyang Xiangcheng (*C. junos Sieb*. ex Tanaka) leaves of plants grown at low concentrations of La (≤1.5 mmol·L^−1^) were similar to those of the control but were significantly reduced at 4 mmol·L^−1^ La. Toxic symptoms gradually appeared with increasing La concentrations, with yellowed leaves and burst veins appearing at 4 mmol·L^−1^ La. The symptoms of toxicity were most severe in trifoliate orange, followed by Shatian Pomelo (*Citrus grandis* var. *shatinyu* Hort) and then Ziyang Xiangcheng. Moreover, in leaves, the Ca content was significantly negatively correlated with La content (*p* < 0.01). These results indicate that La has a hormesis effect on the growth of citrus rootstocks. Of the studied citrus seedlings, Ziyang Xiangcheng is the most resistant to La.

## 1. Introduction

Rare earth elements (REEs) are a homogenous group of 17 chemical elements in the periodic table, and their increasing use in industrial and agricultural practices has resulted in REEs being widely studied in recent years to better understand their environmental effects [1]. Increasing the level of REEs in the soil directly affects the growth and development of plants [2,3]. Numerous studies have revealed that REEs have a hormesis effect on the growth and development of plants [4,5,6,7]. An appropriate amount of REEs is beneficial to plants, promoting plant growth and development as well as improving their photosynthetic capacity. However, high concentrations of REEs may have toxic effects on plants, mainly leading to the slowing of growth, wilting and yellowing leaves, and weakening, as measured by other physiological and biochemical indicators [4,5]. Ce(NH_4_)_2_(NO_3_)_6_ at concentrations of 50 mg·L^−1^ can significantly inhibit the seed germination and root activity of soybeans [6]. At low concentrations, cerium (Ce) has been observed to promote the growth of scallions but is inhibitory at high concentrations, while the chromosome aberration rate increases with the concentration of Ce [7]. In addition, REEs also have a hormesis effect on the structure of the plasma membrane. Low doses of REEs may improve the structural stability of the plasma membrane [8,9], while high doses of REEs may cause changes in the membrane protein structure and increase membrane permeability [10].

China is the country with the largest reserves of rare earth elements (REEs) in the world; the main features of these reserves are their extensive areas and large varieties of REEs, which are widely distributed [11]. REE deposits have been found in 18 provinces and autonomous regions across the country. The main citrus-producing areas in China—Jiangxi, Guangxi, Hubei, Guizhou, and Yunnan—have the highest REE contents [12]. The numerous applications of REEs in modern industry and agriculture will inevitably lead to the accumulation of REEs in the soil, which will affect living organisms, agroecosystems, and the environment [13,14,15].

Citrus is the most economically significant cultivated fruit in southern China. At present, there are 22 provinces (cities, autonomous regions) engaged in citrus production in China, among which Guangxi, Guangdong, Hunan, Hubei, Zhejiang, Fujian, Jiangxi, Sichuan, and Chongqing are the main producing areas [16]. Since REE deposits are geographically coincident with citrus-producing areas and REE fertilizers are universally used in these areas, the content of REEs in citrus orchard soils has gradually increased [17]. Therefore, studying the response of citrus seedlings to REEs is of great economic significance. However, limited studies have been conducted on the effects of REEs on the growth and development of citrus crops. An open question remains as to whether REEs have a hormesis effect on citrus rootstocks.

Since REEs share similar chemical properties, lanthanum (La) has been extensively studied as a representative REE [7,8,18]. Here, La is again used as a representative REE. Trifoliate orange (*Poncirus trifoliata* L. Raf., TO) and Ziyang Xiangcheng (*C. junos Sieb*. ex Tanaka, ZYXC) are the most used rootstocks in China [19]. Shatian pomelo is a kind of high-quality fruit grown in south China that is also used as citrus rootstock [20]. The growth and toxicity indexes of three citrus rootstock seedlings—trifoliate orange (*Poncirus trifoliata* L. Raf., TO), Ziyang Xiangcheng (*C. junos Sieb*. ex Tanaka, ZYXC), and Shatian Pomelo (*Citrus grandis var. shatinyu* Hort, SP)—were compared under different concentrations of La. This study aims to compare the growth and physiological responses of different citrus rootstocks under different concentrations of La, preliminarily selecting the citrus rootstocks with good La tolerance and providing a theoretical basis for citrus cultivation in REE-rich areas.

## 2. Results

### 2.1. Changes in the Growth Parameters of the Three Citrus Rootstocks under La Treatment

**Figure 1:**
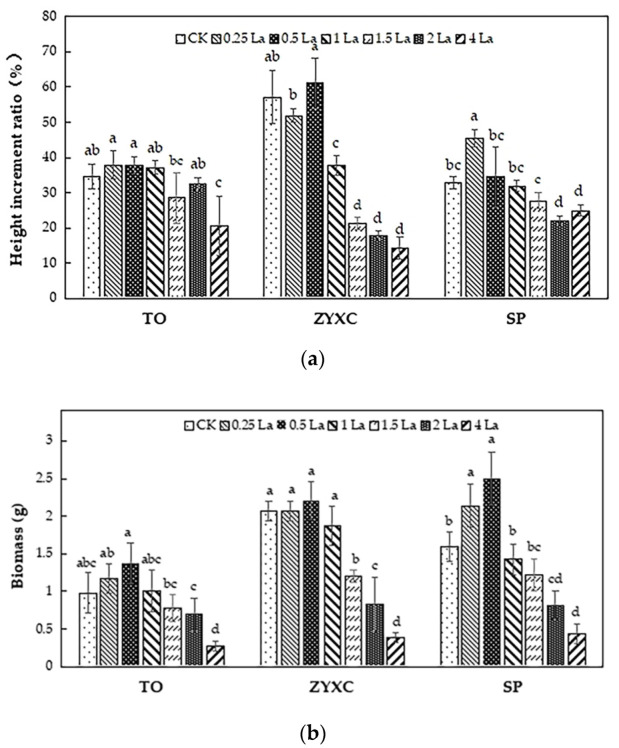
Effects of different La concentrations on growth parameters: (**a**) height increment ratio (HIR); (**b**) biomass of 3 citrus rootstocks. HIR was determined based on the plant height of labeled seedlings at the beginning and end of the treatment, and biomass was measured at the end of the treatment with various concentrations of La (CK = 0 mmol·L^−1^, 0.25 La = 0.25 mmol·L^−1^, 0.5 La = 0.5 mmol·L^−1^, 1 La = 1 mmol·L^−1^, 1.5 La = 1.5 mmol·L^−1^, 2 La = 2 mmol·L^−1^, 4 La = 4 mmol·L^−1^). An ANOVA was performed, and the data are the mean values ± standard errors (SEs) of three biological replicates. Identical letters indicate nonsignificant differences at the 5% probability level according to Duncan’s test. Different letters indicate statistically significant differences with *p* < 0.05. TO = trifoliate orange (*P. trifoliata*); ZYXC = Ziyang Xiangcheng (*C. junos*); SP = Shatian Pomelo (*C. grandis*).

The growth parameters of the three citrus rootstocks differed according to the different La treatments used (Figure 1). In general, the plant height increment ratio of the three citrus rootstocks first increased and then decreased. For TO, when the La content was less than or equal to 2 mmol·L^−1^, the plant height increment ratio was not significantly different from that of the control group. Nevertheless, it was significantly lower than that of the control (*p* < 0.05) when the La concentration was greater than 2 mmol·L^−1^. When the concentration of La was less than or equal to 0.5 mmol·L^−1^, the plant height increment ratio of ZYXC was almost the same as that in the CK group. However, the plant height increment ratio decreased gradually and was significantly lower than that of the control (*p* < 0.05) when the La concentration was greater than 0.5 mmol·L^−1^. For SP, the plant height increment ratio first increased and then decreased with the increase in the La concentration. The maximum height increment ratio was 45.42%, which was observed for the 0.25 mmol·L^−1^ group.

The trends for the biomass of the three citrus rootstocks, which first increased and then decreased with the increase in La concentration, were similar to those of the height increment ratio. The maximum biomass of TO, ZYXC, and SP, which were 1.73, 2.21, and 2.51 g, respectively, occurred under the 0.5 mmol·L^−1^ La treatment. Furthermore, the minimum biomass values (0.28, 0.39, and 0.44 g, respectively) occurred in the 4 La group.

### 2.2. Investigation of Phenotypic Symptoms and Toxicity Indexes in the High-La Treatment

**Figure 2:**
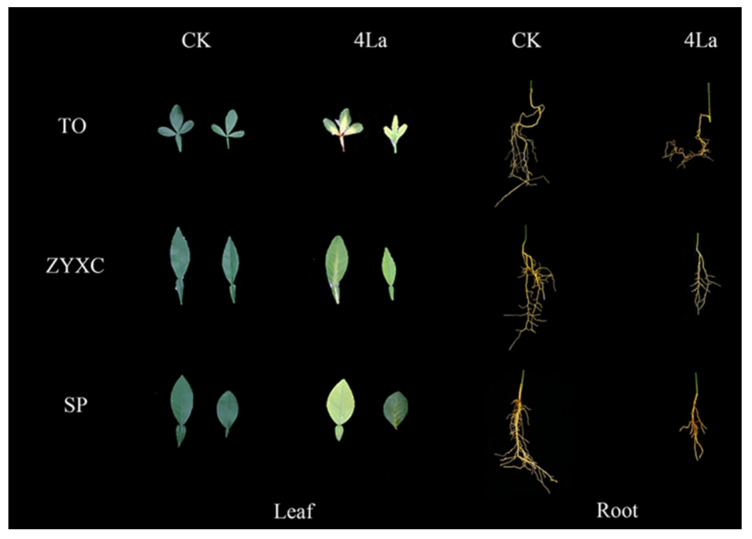
The symptoms and morphologies of the leaves and roots of the 3 rootstocks in the CK (0 mmol·L^−1^) and 4 La (4 mmol·L^−1^) treatments.

In the treatment with the highest concentration of La (4 La), the citrus rootstocks showed obvious symptoms of toxicity. As shown in Figure 2, the leaves of the three citrus rootstocks in the 4 La group showed chlorosis, which is characterized by yellow veins. In more severe cases, whole leaves had yellowed, and the veins had burst. A comparison of the morphological characteristics of the roots of the three rootstocks in the 4 La treatment and control groups (Figure 2) showed that the roots of the plants under the high-concentration treatment were significantly shorter than those in the control group, and the number of lateral roots was significantly lower than those in the control group. This result reveals that root growth is significantly inhibited under high La concentrations.

Throughout the experiment, when the La concentration was lower than 1 mmol·L^−1^, the three citrus rootstocks grew well, showing similar growth to the control, and did not have any symptoms of damage. Therefore, we calculated the damage symptoms and toxicity indexes of the three citrus rootstocks in the 1 La, 1.5 La, 2 La, and 4 La treatment groups. Table 1 shows that in the 1 La treatment group, both TO and SP had Grade 1 toxicity symptoms, while ZYXC did not show any toxicity symptoms until the end of the trial. In a higher-concentration treatment (1.5 La), TO was the first to present Grade 2 toxicity symptoms, followed by SP and ZYXC. Similarly, in the 2 La and 4 La treatment groups, TO was the first rootstock to show toxicity symptoms (Grade 4), followed by SP and ZYXC.

The toxicity index further revealed the difference in the La tolerance of the three citrus rootstocks. In the 1 La treatment group, the toxicity index of ZYXC was 0, but those of TO and SP were 2.08% and 2.50%, respectively. In the 4 La treatment group, the average toxicity indexes of the TO, ZYXC, and SP rootstocks were 50%, 38.33%, and 42.92%, respectively. As the tolerance of citrus rootstocks to La is negatively correlated with the toxicity index, it can be deduced that TO has the highest sensitivity to La, followed by SP, while ZYXC is the most resistant rootstock.

### 2.3. Changes in Chlorophyll and Carotenoid Contents of the Three Citrus Rootstocks’ Leaves under La Treatment

The leaves of the three citrus rootstocks were collected, and their photosynthetic pigment contents were measured. The chlorophyll *a* content of ZYXC in the 4 La treatment group was significantly lower than that of the control group (Table 2). At La ≥ 2 mmol·L^−1^, the chlorophyll *a* content of TO was significantly lower than that of the control group. For SP, at La ≥ 0.5 mmol·L^−1^, the chlorophyll *a* content was significantly lower than that of the control group (Table 2). The chlorophyll *b* content of ZYXC and TO significantly decreased under the 4 La treatment, and the chlorophyll *b* content of SP was significantly lower than that of the control group at La ≥ 0.5 mmol·L^−1^. The changing trend of chlorophyll *a* + *b* in the three citrus rootstocks was similar to that of chlorophyll *a*, while the changing trend of carotenoids was consistent with that of chlorophyll *b* (Table 2).

### 2.4. Changes in MDA Content and Antioxidant Enzyme Activities of the Three Citrus Rootstocks’ Leaves under La Treatment

The malondialdehyde contents of leaves of the three citrus rootstocks first decreased and then increased with increasing concentrations of La, with the lowest levels observed for 1 La out of all the treatments(Table 3). These values were significantly higher than the control when the concentration of La was 4 mmol·L^−1^. The SOD activity of ZYXC and TO first increased and then decreased with the increase in La concentration. The SOD activity of SP was reduced to varying degrees under the La treatment(Table 3). The SOD activity of ZYXC under different concentrations of La showed no significant difference from that of the control group, and the maximum was 211.99 U·g^−1^·min^−1^ FW under the 0.5 La treatment. The SOD activity of TO and SP was significantly lower under treatment with concentrations ≥0.5 mmol·L^−1^ La than that of the control group. The CAT activity of the leaves of the three rootstocks first increased and then decreased with the increase in La concentration and reached a maximum at 0.5 mmol·L^−1^ La(Table 3).

### 2.5. La Absorption and Transport in Citrus Rootstocks

**Figure 3:**
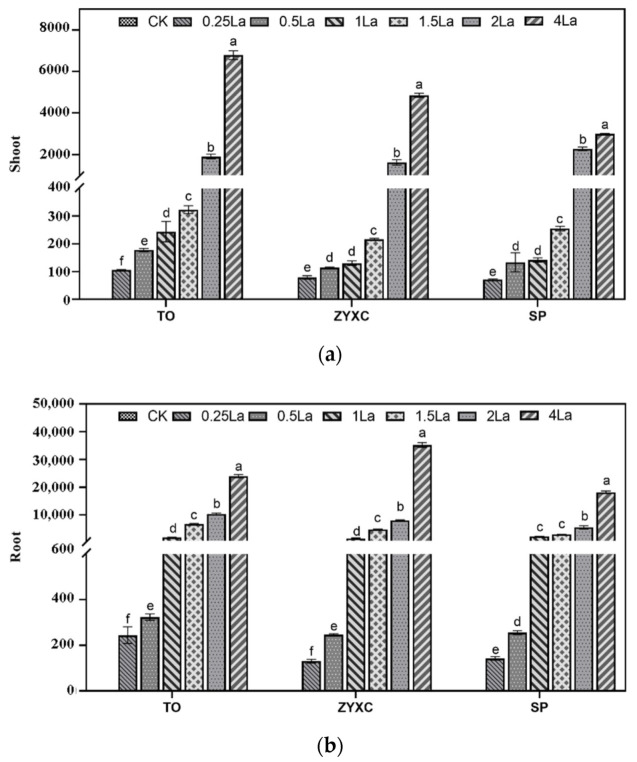
Distribution of La in the (**a**) shoots and (**b**) roots of the 3 citrus rootstock seedlings (mg/kg). An ANOVA was performed. Identical letters indicate nonsignificant differences at the 5% probability level based on Duncan’s test. Different letters indicate statistically significant differences, with *p* < 0.05. Data are shown as mean ± SE (*n* = 3, all leaves of 3 plants). TO = Trifoliate orange (*P. trifoliata*), ZYXC = Ziyang Xiangcheng (*C. junos*), SP = Shatian Pomelo (*C. grandis*). The three citrus rootstocks had no distribution (ND) of La in the CK treatment (0 mmol·L^−1^); 0.25 La = 0.25 mmol·L^−1^ La, 0.5 La = 0.5 mmol·L^−1^ La, 1 La = 1 mmol·L^−1^ La, 1.5 La = 1.5 mmol·L^−1^ La, 2 La = 2 mmol·L^−1^ La, 4 La = 4 mmol·L^−1^ La.

The distribution of La in the aboveground and belowground parts of the three citrus rootstocks under different treatments is shown in Figure 3. The La contents in both the shoots and roots of the three rootstocks increased with the increasing La concentration. Additionally, the content of La in the roots was higher than that in the shoots for each rootstock.

The La migration coefficients of the three rootstocks under different La treatments are shown in Table 4. With the increase in La concentration, the migration coefficients of ZYXC and SP first decreased and then increased and peaked in the 0.25 La treatment, with values of 25.65% and 22.24%, respectively. TO reached a maximum migration coefficient value (14.76%) in the 4 La treatment group. Overall, ZYXC had the highest values for La migration coefficients, followed by SP and TO.

### 2.6. Effect of La on the Calcium Content in the Citrus Leaves

**Figure 4:**
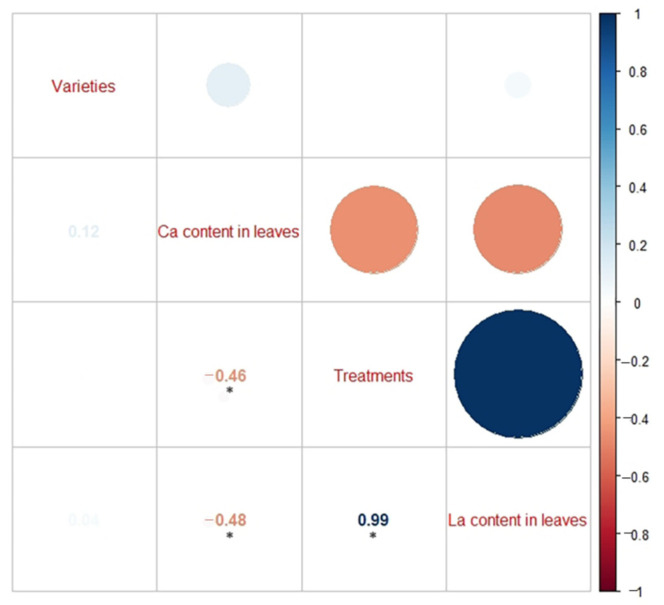
Correlation between the La content and the Ca content in the citrus rootstock leaves under the La treatments. The results showed the following: (1) the Ca content in the leaves was significantly negatively correlated with the La content, and (2) the La content in the leaves was significantly correlated with the La concentrations under the different treatments.

Under different La treatments, the Ca content in the leaves of the three citrus rootstocks generally decreased (Table 5). The Spearman correlation coefficient between the Ca and La contents in the three citrus rootstocks’ leaves was −0.48, indicating that the Ca content in the leaves was significantly negatively correlated with the La content (Figure 4).

## 3. Discussion

### 3.1. Effect of La Stress on the Growth of Three Citrus Rootstocks

Previous studies have shown that REEs can significantly regulate the growth and development of plants [21,22,23,24,25]. REEs enhance plant biomass by stimulating the uptake of mineral nutrients and promoting the synthesis of chlorophyll [21]. La increases crop yield by modulating the activity of RuBP carboxylase [22]. However, many studies have shown that the promotion of plant growth and development occurs under low concentrations of REEs, and an inhibitory effect has been observed under high concentrations [4,7,23]. An appropriate concentration of La^3+^ had a promoting effect on the germination rate and germination potential of *Salvia miltiorrhiza* seeds, and the promotion effect was highest at 30 mg·L^−1^ La^3+^. Meanwhile, the soluble sugar and soluble protein contents and the activity of the antioxidant enzyme system were improved, showing increases. In contrast, La^3+^ could inhibit the growth of the plants at high concentrations [24]. Circulation of the appropriate amount of REEs during plant growth has been previously observed to promote plant growth and development [25]. Similarly, we found that the three rootstocks did not show any symptoms of damage and that the height increment ratio and biomass of the plants increased, to some extent, when the La concentration was less than or equal to 0.5 mmol·L^−1^. However, when the La content exceeded 0.5 mmol·L^−1^, the height increment ratio and biomass of the three rootstocks were reduced and significantly lower than those of the control group. Moreover, the La migration coefficients of ZYXC and SP first decreased and then increased with increasing La concentrations. These results indicate that REEs have a hormesis effect on plant growth and development.

A large number of studies have shown that REEs have a hormesis effect on the growth and development of plants [26,27]. Appropriate concentrations of La (5–10 mg·L^−1^) and Ce (5–20 mg·L^−1^) can effectively increase the antioxidant enzyme activity of pea seedlings and reduce the MDA content and significantly reduce the toxic effect of Cu stress (*p* < 0.05). However, high concentrations of the two REEs will aggravate the damage to the antioxidant enzyme system of pea seedlings caused by Cu, showing a collaborative toxic effect [26]. Ce(NH_4_)_2_(NO_3_)_6_ solutions promoted root growth and increase the chlorophyll content under treatments of 1 and 10 mg·L^−1^, while 30 and 50 mg·L^−1^ Ce(NH_4_)_2_(NO_3_)_6_ solutions were inhibitory [27]. We found that none of the three rootstocks showed toxicity symptoms under a low concentration of La (0.5 mmol·L^−1^), while the three rootstocks showed varying degrees of toxicity symptoms under treatments of a high concentration of La (≥1 mmol·L^−1^). During the test, the earliest toxicity symptoms and the highest average value for the toxicity index were observed in TO, while ZYXC was the last rootstock to show toxicity symptoms, and it also had the lowest average value for the toxicity index. These findings indicate that of the studied rootstocks, TO is the most sensitive to La, followed by SP, and ZYXC is the most resistant.

The chloroplast is important for green plants, being the site where photosynthesis occurs. Various photosynthetic pigments that can absorb, transmit, and transform light energy are distributed on the thylakoid membrane of the chloroplast, including the majority of chlorophyll *a*, which collects and transmits light energy, all of chlorophyll *b*, and carotenoids. A few special chlorophyll *a* molecules directly contribute to the conversion of light energy into chemical energy [28]. A large number of studies have demonstrated that REEs can affect the chlorophyll content of plants. Treatment of the green algae *Chlorella* with low concentrations of the rare earth element Eu resulted in increases in the chlorophyll *a* and *b* contents to levels significantly higher than those of the control. A high concentration of Eu resulted in chlorophyll reduction and the inhibition of *Chlorella* growth [29]. The chlorophyll content and photosynthetic rate of *Lonicera japonica* increased when treated with low concentrations of La and decreased when the concentration of La was above 30 mg∙L^−1^ [30]. Our study found that under low concentrations of La (2 mmol∙L^−1^), the contents of chlorophyll *a*, chlorophyll *b*, chlorophyll *a* + *b*, and carotenoids in leaves of the three citrus rootstocks were similar to those of the control group. However, the chlorophyll and carotenoid contents of the three rootstocks were all significantly reduced under the 4 La treatment, which is consistent with the toxic symptoms observed in Figure 2.

### 3.2. Effects of La on Physiological and Biochemical Indexes of Three Citrus Rootstocks

MDA is a lipid peroxidation product of cell membranes, and its content can, to an extent, reflect the degree of oxidative damage to plants [31]. Previous studies have shown that low concentrations of La can effectively reduce the contents of MDA and H_2_O_2_ in *Lonicera japonica* leaves, and high concentrations of La will increase the content of MDA and H_2_O_2_ in the leaves. Similar results were obtained in research on horseradish and *Dendrobium hancockii* [32]. The results of our study showed that the MDA contents of the three citrus rootstocks’ leaves first decreased and then increased with increasing concentrations of La, but they were significantly higher than the control at a high concentration of 4 mmol∙L^−1^.

During the growth and development of plants, a large number of free radicals are produced that peroxidize membrane lipids, destroy the membrane system, and even cause cell death [33]. SOD and CAT are the main enzymes of the plant antioxidant system that remove reactive oxygen species [23]. REEs also have a significant impact on the active oxygen metabolism system of plants. In a study of *Arabidopsis*, it was found that a low concentration of La^3+^ can enhance the enzyme activity of SOD [34]. A 20–30 mg∙L^−1^ La^3+^ treatment significantly increased the SOD and CAT enzyme activities of longan leaves while improving the efficiency of the AsA-GSH cycle and the activity of related metabolic enzymes, thereby improving the La resistance of longan [35]. Our study found that the SOD activity of ZYXC and TO first increased and then decreased with the increase in La concentration. The SOD activity of ZYXC under different concentrations of La was not significantly different from that of the control group. The SOD activity of TO and SP under La treatment was significantly lower than that of the control group. The CAT activity of the three rootstocks’ leaves first increased and then decreased with the increase in La concentration, reaching a maximum at 0.5 mmol·L^−1^ La.

### 3.3. Effect of La on the Ca Content of the Three Citrus Rootstocks

Previous studies have found that the REE content was highest in the roots of plants, accounting for approximately 80%, followed by the stems, leaves, flowers, fruits, and seeds [36,37]. The distribution of five REEs (La, Ce, Nd, Y, Gd) and single REEs in crops has been previously studied, and the absorption capacity of crops for REEs has been found to be positively correlated with the amount available in the soil. The order of the distribution of the REE content in each part of the plant is generally root > leaf > stem > fruit [38]. La is referred to as super calcium. La^3+^ often occupies the position of Ca^2+^ and binds to biomacromolecules, even replacing bound Ca^2+^, which interferes with the normal physiological functioning of Ca^2+^ [39,40]. Additionally, La^3+^ binds to the site of Ca^2+^ on the plasma membrane, which affects the absorption and transportation of Ca^2+^; therefore, La^3+^ is considered an inhibitor of Ca^2+^ channels [41]. In this study, under different La treatments, the La content in the roots of the three citrus rootstock seedlings was found to be significantly higher than that in the shoots, which is consistent with the results of previous studies. We also found that the Ca content in the leaves of the rootstocks was significantly negatively correlated with the La content (*p* < 0.01). The correlation coefficient was −0.48, which indicates that high concentrations of La would affect the absorption of calcium by plants. These results indicate that La has a hormesis effect on the growth of citrus rootstocks. With the increase in La concentration, the order of La tolerance of the three citrus rootstocks is Ziyang Xiangcheng > Shatian Pomelo > trifoliate orange. Hence, our results also show that La treatment affects the absorption of calcium in citrus rootstocks.

## 4. Materials and Methods

### 4.1. Plant Materials and Treatments

Three main types of citrus rootstocks in China were used in this study: trifoliate orange (*Poncirus trifoliata* L. Raf., TO), Ziyang Xiangcheng (*C. junos Sieb*. ex Tanaka, ZYXC), and Shatian Pomelo (*Citrus grandis* var. *shatinyu* Hort, SP). The culture of rootstock seedlings can be divided into three stages. At the first germination stage, the seeds with plump and consistent grains were selected and soaked in distilled water for 24 h. Then, the seed coats were peeled off. After soaking in 0.1% potassium permanganate solution for 3–5 min, the seeds were repeatedly cleaned with distilled water and placed on a floating plate in a plastic box filled with distilled water. The seeds were cultured in an incubator in the dark for 3 to 5 days. The second stage was the cultivation period of rootstock seedlings. From the rooting of the plants to the growth period of 3 to 5 true leaves, the plants were planted in a sandy medium (quartz sand:Perlite = 1:1) and then placed in the culture room, with conditions of 5000 LX light intensity, 12 h/Day, and a day/night temperature of 25/18 °C. Distilled water (50 mL) was poured on the plants once every 2 to 3 days. The third stage was the nutrient culture stage. After 3 to 5 true leaves sprouted from the rootstock seedlings, the culture was gradually adjusted from distilled water to modified Hoagland solution [42], and the rest of the culture conditions remained unchanged.

After two months, the seedlings were treated with La(NO_3_)_3_·6H_2_O solution at concentrations of 0 (CK), 0.25 (0.25 La), 0.5 (0.5 La), 1 (1 La), 1.5 (1.5 La), 2 (2 La), and 4 mmol·L^−1^ (4 La). Three replicates were used, with 10 seedlings per variety and replicate. Each labeled pot was watered with a corresponding concentration of treatment solution (400 mL) every three days. The experiment was terminated when more than 50% of the rootstocks had died in the highest concentration treatment.

### 4.2. Determination of the Growth Parameters

Fifteen seedlings of each treatment were selected and marked. The plant height of the labeled seedlings was measured at the beginning and end of the treatment to determine the height increment ratio. At the end of the experiment, whole seedlings were collected from each treatment. Subsequently, the biomass of the samples was measured by electronic scales.

$$\mathrm{Height}\;\mathrm{increment}\;\mathrm{ratio}=\frac{\mathrm{measured}\;\mathrm{value}\;\mathrm{after}\;\mathrm{treating}\;-\;\mathrm{measured}\;\mathrm{value}\;\mathrm{before}\;\mathrm{treating}}{\mathrm{measured}\;\mathrm{value}\;\mathrm{before}\;\mathrm{treating}}\times 100\%$$

### 4.3. Investigation of Phenotypic Symptoms and Toxicity Indexes

Changes in the morphology and color of the rootstock leaves were observed daily, and the date of symptom onset, the corresponding toxicity grade, and the displayed symptoms were recorded. Toxicity grades were assigned according to the salt damage grading standard and the morphological changes in the citrus rootstocks under La stress [43]. Grade 0 indicates that there is no significant difference between the leaves of the treatment group and those of the control group and that no damage symptoms are visible. In Grade 1, the leaves are mildly damaged, with yellow leaves accounting for 10–25% of all the leaves on the plant. Grade 2, moderate toxicity is characterized by yellow leaves covering 25–50% of the plant. In Grade 3, the severely toxic stage, burst veins or symptoms of dehydration are observed, and yellow leaves cover 50 to 75% of the whole plant. In Grade 4, extremely severe poisoning, more than 75% of the leaves have yellowed, the leaves are dry, and the branches are dead.

$$\mathrm{Toxicity}\;\mathrm{index}\;\left(D\right)=\frac{\sum \left(\mathrm{toxicity}\;\mathrm{grade}\;\times \;\mathrm{number}\;\mathrm{of}\;\mathrm{plants}\;\mathrm{in}\;a\;\mathrm{grade}\right)}{\mathrm{total}\;\mathrm{number}\;\mathrm{of}\;\mathrm{plants}\;\mathrm{investigated}\;\times \mathrm{highest}\;\mathrm{toxicity}\;\mathrm{grade}}\times 100\%$$

### 4.4. Determination of Photosynthetic Pigment Content in Leaves of Citrus Rootstock

Our method was based on a measurement method used in a previous study [44]. Firstly, the leaves without the main veins were cut into small pieces, weighed to 0.1 g accurately, and used for extraction with 10 mL of 96% ethanol. Secondly, the mixed liquor was placed in the dark for 24 h; then, it was mixed well, and the supernatant was collected. Finally, the OD values at 665, 649, and 470 nm were read using a TU-9101 UV spectrophotometer and used to calculate the contents of chlorophyll a, b, and a + b and carotenoids.

$$\mathrm{The}\;\mathrm{content}\;\mathrm{of}\;\mathrm{Chlorophyll}\;a\;\left(\mathrm{Chla}\right)\;\left(\mathrm{mg}/g\right)=\frac{\left(13.95\times {\mathrm{OD}}_{665}-6.88\times {\mathrm{OD}}_{649}\right)\times V}{W}\times 10^{-3}$$

$$\mathrm{The}\;\mathrm{content}\;\mathrm{of}\;\mathrm{Chlorophyll}\;b\;\left(\mathrm{Chlb}\right)\;\left(\mathrm{mg}/g\right)=\frac{\left(24.96\times {\mathrm{OD}}_{649}-7.32\times {\mathrm{OD}}_{665}\right)\times V}{W}\times 10^{-3}$$

The content of Chlorophyll a+b (Chl a+b) (mg/g)=Chla+Chlb

$$\mathrm{The}\;\mathrm{content}\;\mathrm{of}\;\mathrm{Carotenoids}\;\left(\mathrm{mg}/g\right)=\frac{\left(1000\times {\mathrm{OD}}_{470}-2.05\mathrm{Chla}-114.8\mathrm{Chlb}\right)\times V}{W\times 245}\times 10^{-3}$$

V: volume of the chlorophyll extract; W: fresh weight of extracted leaves (g).

### 4.5. Determination of Physiological and Biochemical Indicators of Citrus Rootstock Leaves

We took the last 3–5 fully expanded leaves of each treatment on the 7th day after treatment and quickly froze them with liquid nitrogen and stored them in a −80 °C freezer until they were used for testing. The content of malondialdehyde (MDA) and the activities of superoxide dismutase (SOD) and catalase (CAT) were measured using a kit provided by Suzhou Comin Biotechnology Co., Ltd. (Suzhou, China) (MDA: MDA-2-Y; SOD: SOD-2-Y; CAT: CAT-2-W). We followed the manufacturer’s specific instructions that were included with the kit.

### 4.6. Determination of La and Ca contents

At the end of the experiment, the seedling shoots and roots of each treatment were harvested separately, with three replicates. The shoots and roots were digested using the electrothermal plate method [45], and the La content was determined using an Agilent 5110 (Agilent, Santa Clara, CA, USA) inductively coupled plasma optical emission spectrometer (ICP-OES). Standard curve: using a stock solution of 100 μg/mL La, we prepared solutions of 1, 2, 5, 8, and 10 μg/mL by dilution, using HNO_3_ as the medium. Instrument settings: RF transmitter power 1000 W, carrier gas flow 15 L/min, auxiliary gas flow 0.2 L/min, atomizer flow 0.8 L/min, peristaltic pump speed 1.5 mL/min, axial observation, repeat 3 times. The Ca content in the plant leaves was determined using an AA-800 atomic absorption spectrometer (PerkinElmer, Waltham, MA, USA) [46].

$$\mathrm{La}\;\mathrm{migration}\;\mathrm{coefficients}=\frac{\mathrm{La}\;\mathrm{content}\;\mathrm{in}\;\mathrm{shoots}}{\mathrm{La}\;\mathrm{content}\;\mathrm{in}\;\mathrm{shoots}+\mathrm{La}\;\mathrm{content}\;\mathrm{in}\;\mathrm{roots}}\times 100\%$$

## 5. Conclusions

(1) La has a hormesis effect on the growth of citrus rootstocks. When the La concentration is less than or equal to 0.5 mmol·L^−1^, the growth of citrus rootstock seedlings is stimulated. However, when the La content is higher than 1 mmol·L^−1^, citrus rootstock growth is inhibited.
(2) TO is the rootstock most sensitive to La, followed by SP. ZYXC is the most resistant rootstock.
(3) The accumulation of La in the roots is higher than that in the shoots of the rootstocks, and the content increases with an increasing La concentration.
(4) The La treatments affect the absorption of calcium by citrus rootstocks.

## Figures and Tables

**Table 1:** Initial time of the occurrence of La injury symptoms in the 3 citrus rootstock seedlings.

Treatment	Variety	Time to the Occurrence of Injury Symptoms (Days)				Toxicity Index (%)
		Grade 1	Grade 2	Grade 3	Grade 4	
1 La	TO	32	—	—	—	2.08
	ZYXC	—	—	—	—	0
	SP	34	—	—	—	2.5
1.5 La	TO	30	40	—	—	2.92
	ZYXC	31	43	—	—	2.92
	SP	29	43	—	—	3.75
2 La	TO	20	22	25	30	22.5
	ZYXC	24	27	34	43	17.08
	SP	22	25	27	41	20
4 La	TO	14	16	22	28	50
	ZYXC	16	21	27	37	38.33
	SP	14	18	24	32	42.92

Note: TO = Trifoliate orange (*P. trifoliate*), ZYXC = Ziyang Xiangcheng (*C. junos*), SP = Shatian Pomelo (*C. grandis*). 1 La = 1 mmol·L^−1^ La, 1.5 La = 1.5 mmol·L^−1^ La, 2 La = 2 mmol·L^−1^ La, 4 La = mmol·L^−1^ La. (*n* = 3).

**Table 2:** Effects of La on the chlorophyll and carotenoid contents in the leaves of the 3 citrus rootstocks.

Rootstocks	Treatments	Chlorophyll *a* (mg·g^−1^, FW)	Chlorophyll *b* (mg·g^−1^, FW)	Chlorophyll *a* + *b* (mg·g^−1^, FW)	Carotenoids (mg·g^−1^, FW)
ZYXC	CK	2.19 ± 0.03 b	0.86 ± 0.04 b	3.04 ± 0.06 b	0.77 ± 0.02 c
	0.25 La	2.39 ± 0.04 a	1.02 ± 0.05 a	3.41 ± 0.09 a	0.86 ± 0.02 a
	0.5 La	2.44 ± 0.08 a	0.97 ± 0.04 a	3.41 ± 0.12 a	0.72 ± 0.01 ab
	1 La	2.44 ± 0.05 a	0.98 ± 0.04 a	3.42 ± 0.09 a	0.83 ± 0.02 ab
	1.5 La	2.48 ± 0.03 a	1.04 ± 0.02 a	3.52 ± 0.04 a	0.87 ± 0.01 a
	2 La	2.2 ± 0.17 b	0.86 ± 0.08 b	3.05 ± 0.25 b	0.79 ± 0.05 bc
	4 La	1.39 ± 0.13 c	0.55 ± 0.05 c	1.94 ± 0.18 c	0.57 ± 0.05 d
TO	CK	3.15 ± 0.05 a	1.37 ± 0.04 ab	4.52 ± 0.09 a	1.01 ± 0.03 a
	0.25 La	2.61 ± 0 b	1.2 ± 0.12 ab	3.88 ± 0.03 cd	0.85 ± 0.06 bc
	0.5 La	2.86 ± 0.45 ab	1.21 ± 0.16 b	4.38 ± 0.3 ab	0.89 ± 0.13 ab
	1 La	2.71 ± 0.15 b	1.25 ± 0.13 ab	3.8 ± 0.06 cd	0.87 ± 0.05 bc
	1.5 La	2.94 ± 0.15 ab	1.44 ± 0.14 a	4.38 ± 0.28 ab	0.93 ± 0.05 ab
	2 La	2.77 ± 0.21 b	1.34 ± 0.11 ab	4.11 ± 0.32 bc	0.92 ± 0.06 ab
	4 La	2.59 ± 0.1 b	0.92 ± 0.04 c	3.51 ± 0.14 d	0.77 ± 0.03 c
SP	CK	3.02 ± 0.01 a	1.53 ± 0.14 a	4.55 ± 0.15 a	1.07 ± 0.03 a
	0.25 La	2.88 ± 0.17 ab	1.33 ± 0.02 ab	4.21 ± 0.18 ab	1.01 ± 0.05 ab
	0.5 La	2.6 ± 0.06 bc	1.19 ± 0.05 b	3.79 ± 0.11 bc	0.92 ± 0.02 bc
	1 La	2.41 ± 0.57 cd	0.98 ± 0.28 c	3.39 ± 0.85 cd	0.76 ± 0.02 cd
	1.5 La	2.34 ± 0.1 cd	0.95 ± 0.08 c	3.29 ± 0.18 cd	0.84 ± 0.04 cd
	2 La	2.19 ± 0.04 d	0.86 ± 0.03 c	3.05 ± 0.08 d	0.77 ± 0.02 d
	4 La	1.07 ± 0.01 e	0.46 ± 0.01 d	1.53 ± 0.02 e	0.45 ± 0.01 e

Note: An ANOVA was performed. Identical letters indicate nonsignificant differences at the 5% probability level based on Duncan’s test. Different letters indicate statistically significant differences with *p* < 0.05. Data are shown as mean ± SE (*n* = 3: all leaves of 3 plants). TO = Trifoliate orange (*P. trifoliata*), ZYXC = Ziyang Xiangcheng (*C. junos*), SP = Shatianyou (*C. grandis*). The three citrus rootstocks had no distribution (ND) of La in the CK treatment (0 mmol·L^−1^); 0.25 La = 0.25 mmol·L^−1^ La, 0.5 La = 0.5 mmol·L^−1^ La, 1 La = 1 mmol·L^−1^ La, 1.5 La = 1.5 mmol·L^−1^ La, 2 La = 2 mmol·L^−1^ La, 4 La = 4 mmol·L^−1^ La.

**Table 3:** Effects of La on the antioxidant enzyme activity and MDA content in the leaves of citrus rootstock seedlings.

Rootstocks	Treatments	MDA (nmol·g^−1^ FW)	SOD (U·g^−1^·min^−1^ FW)	CAT (U·g^−1^·min^−1^ FW)
ZYXC	CK	22.74 ± 0.99 c	198.54 ± 84.76 a	106.6 ± 1.06 c
	0.25 La	24.78 ± 3.44 bc	202.03 ± 31.01 a	301.11 ± 112.1 b
	0.5 La	25.55 ± 1.46 bc	211.99 ± 64.4 a	478.44 ± 31.59 a
	1 La	22.99 ± 1.62 c	202.11 ± 19.45 a	251.45 ± 22.02 b
	1.5 La	26.4 ± 1.96 bc	200.21 ± 14.76 a	250.25 ± 54.53 b
	2 La	31.49 ± 4.96 ab	188.44 ± 8.16 a	207.98 ± 20.33 bc
	4 La	35.8 ± 6.99 a	132.97 ± 8.89 a	172.39 ± 100.78 bc
TO	CK	89.88 ± 7.42 bc	409.47 ± 47.84 a	130.19 ± 28.63 c
	0.25 La	66.93 ± 19.46 d	143.43 ± 27.72 c	297.05 ± 57 ab
	0.5 La	72.87 ± 0.41 cd	265.58 ± 16.03 b	352.88 ± 45.56 a
	1 La	61.01 ± 1.31 d	262.45 ± 0.75 b	233.93 ± 9.65 b
	1.5 La	65.85 ± 4.98 d	169.16 ± 34.87 c	291.39 ± 9.28 ab
	2 La	94.63 ± 1.04 b	196.18 ± 53.71 bc	143.02 ± 4.35 c
	4 La	193.41 ± 7.62 a	146.25 ± 20.64 c	128.56 ± 22.89 c
SP	CK	22.13 ± 2.74 b	280.21 ± 21.81 a	37.99 ± 12.39 d
	0.25 La	19.29 ± 0.91 bc	214.33 ± 2.26 ab	140.29 ± 16.03 b
	0.5 La	20.92 ± 1.86 b	165.87 ± 11.06 bc	198.99 ± 5.96 a
	1 La	14.14 ± 2.43 d	135.97 ± 30.45 c	149.07 ± 43.41 b
	1.5 La	15.29 ± 2.13 cd	144.59 ± 24.36 bc	95 ± 6.14 c
	2 La	22.45 ± 3.08 b	141.79 ± 76.14 bc	68.92 ± 12.15 cd
	4 La	37.01 ± 2.52 a	128.39 ± 2.21 c	44.96 ± 12.29 d

Note: An ANOVA was performed. Identical letters indicate nonsignificant differences at the 5% probability level based on Duncan’s test. Different letters indicate statistically significant differences with *p* < 0.05. Data are shown as mean ± SE (*n* = 3: all leaves of 3 plants). TO = Trifoliate orange (*P. trifoliata*), ZYXC = Ziyang Xiangcheng (*C. junos*), SP = Shatianyou (*C. grandis*). The three citrus rootstocks had no distribution (ND) of La in the CK treatment (0 mmol·L^−1^); 0.25 La = 0.25 mmol·L^−1^ La, 0.5 La = 0.5 mmol·L^−1^ La, 1 La = 1 mmol·L^−1^ La, 1.5 La = 1.5 mmol·L^−1^ La, 2 La = 2 mmol·L^−1^ La, 4 La = 4 mmol·L^−1^ La.

**Table 4:** Effects of La treatments on the La migration coefficients of the 3 citrus rootstock seedlings (%).

Rootstock	0.25 La	0.5 La	1 La	1.5 La	2 La	4 La	Mean
TO	5.29	12.73	5.80	3.16	4.74	14.76	7.75
ZYXC	25.65	17.82	7.55	6.09	9.17	14.80	13.51
SP	22.24	14.78	5.13	5.17	5.22	12.94	10.91

Note: La migration coefficients = (La content in shoots)/(La content in shoots + La content in roots) × 100%.

**Table 5:** Effects of La treatments on Ca content in the leaves of three citrus rootstock seedlings (mg/g).

Treatment	ZYXC	TO	SP
CK	31.42 ± 0.55 a	13.53 ± 0.15 c	31.35 ± 1.73 a
0.25 La	24.54 ± 2.71 bc	19.25 ± 0.77 a	26.54 ± 3.31 b
0.5 La	26.28 ± 0.28 bc	16.29 ± 0.69 b	18.17 ± 0.67 d
1 La	27.12 ± 2.2 b	16.33 ± 0.57 b	23.08 ± 1.89 c
1.5 La	20.86 ± 2.19 d	13.61 ± 0.14 c	16.79 ± 2.2 d
2 La	18.76 ± 0.79 de	12.1 ± 0.4 d	18.83 ± 1.64 d
4 La	17.3 ± 0.31 e	13.99 ± 0.38 c	18.81 ± 1 d

Note: An ANOVA was performed. Identical letters indicate nonsignificant differences at the 5% probability level based on Duncan’s test. Different letters indicate statistically significant differences with *p* < 0.05. Data are shown as mean ± SE (*n* = 3, all leaves of 3 plants). TO = Trifoliate orange (*P. trifoliata*), ZYXC = Ziyang Xiangcheng (C. junos), SP = Shatian Pomelo (C. grandis). The three citrus rootstocks had no distribution (ND) of La in the CK treatment (0 mmol·L−1); 0.25 La = 0.25 mmol·L^−1^ La, 0.5 La = 0.5 mmol·L^−1^ La, 1 La = 1 mmol·L^−1^ La, 1.5 La = 1.5 mmol·L^−1^ La, 2 La = 2 mmol·L^−1^ La, 4 La = 4 mmol·L^−1^ La.

## Data Availability

All data are contained within the article.
